# Dr. Richard Bright

**Published:** 1859-03

**Authors:** 


DR. RICHARD BRIGHT.—During the past year, death struck down some of the brightest ornaments that adorned the medical world of Europe, and we have now to record a loss which will be felt by the whole world, to whom its subject was so well known. Dr. Bright, familiar to every one by his writings, more especially upon the connection between disease of the kidney and abdominal dropsy, died in London, in the seventieth year of his age, on the 15th of December, after an illness of five days, of ossification of the aortic valves, which amounted to almost complete closure. The disease was suspected for several years, but as no examination was ever allowed, the extent of involvement was not known until an autopsy was made. Dr. Bright graduated at Edinburgh, in 1813, his thesis being on contagious erysipelas; and his desire for knowledge caused him to seek the great cities of Europe, to gain instruction in the various branches of medicine and surgery. Early in life he evinced a great taste for pathological pursuits, of which he made good use in his works and writings. In 1824 he was appointed Physician to Guy's Hospital, having previously filled the post of Assistant Physician. His contributions to medical periodical literature are very extensive and valuable, and are for the most part to be found in "Guy's Hospital Reports" and the "Transactions of the Medico-Chirurgical Society." In connection with Dr. Addison, he began a text-book, entitled "Elements of the Practice of Medicine," only one volume of which ever appeared. His "Reports of Medical Cases," in two royal quarto volumes, contain much valuable information on the pathology of fever, as well as of the kidneys, lungs, cerebral, spinal and other nervous affections. The first of these appeared in 1827, the other four years subsequently. Dr. Bright was undoubtedly the most prominent British physician of his day, and has left his name connected with granular disease of the kidney, the importance of which affection he was the first to point out. At the time of his decease he was Physician Extraordinary to the Queen.

